# Alterations in MicroRNA and Cytokine Expressions in Placental and Amniotic Tissues of COVID‐19 Affected Pregnant Women

**DOI:** 10.1002/kjm2.70207

**Published:** 2026-04-02

**Authors:** Wei‐Chun Chen, Shu‐Yu Hu, Chao‐Min Cheng, Ching‐Ju Shen

**Affiliations:** ^1^ Institute of Biomedical Engineering National Tsing Hua University Hsinchu Taiwan; ^2^ Division of Gynecologic Oncology, Department of Obstetrics and Gynecology Chang Gung Memorial Hospital at Linkou, College of Medicine Chang Gung University Taoyuan Taiwan; ^3^ Department of Obstetrics and Gynecology New Taipei City Municipal Tucheng Hospital New Taipei City Taiwan; ^4^ International Intercollegiate Ph.D. Program National Tsing Hua University Hsinchu Taiwan; ^5^ School of Traditional Chinese Medicine Chang Gung University Taoyuan Taiwan; ^6^ Department of Obstetrics and Gynecology Kaohsiung Medical University Hospital, Kaohsiung Medical University Kaohsiung Taiwan

**Keywords:** COVID‐19, cytokines array, microRNA, placenta, pregnancy women

## Abstract

Since 2019, coronavirus disease 2019 (COVID‐19) has been associated with increased risks of preterm birth and placental complications. We prospectively investigated alterations in microRNAs (miRNAs) and cytokines in placental and amniotic tissues from pregnant women with and without COVID‐19 to evaluate the infection's impact on pregnancy. Placental and amniotic samples were collected at delivery from 15 pregnant women, including 7 with confirmed COVID‐19 and 8 healthy controls. Tissue lysates were prepared for miRNA sequencing with QIAseq and cytokine profiling using Bio‐Plex ProTM assays, and clinical data were recorded for correlation analyses. We identified three miRNAs, including hsa‐miR‐20a‐5p, hsa‐miR‐20b‐5p, and hsa‐miR‐25‐3p were consistently upregulated in both placental and amniotic tissues following maternal COVID‐19 infection. Eight cytokines, including IL‐4, IL‐9, and IL‐10, showed strong correlations with these miRNA changes. Besides, placental hsa‐miR‐20b‐5p expression was positively correlated with IL‐9 levels. Although expression of hsa‐miR‐142‐3p and hsa‐miR‐155‐5p did not differ significantly between COVID‐19‐positive and control groups, these miRNAs demonstrated inverse correlations with IL‐4 and IL‐10 levels in amniotic samples, suggesting potential immunoregulatory roles during infection. These findings highlight a set of miRNAs with shared regulatory patterns across placental and amniotic compartments and their close association with key cytokines, suggesting common immune mechanisms at the maternal–fetal interface. miRNA–cytokine signatures may serve as candidate biomarkers for monitoring the effects of COVID‐19 in prenatal and postnatal care.

**Abbreviations**
Basic‐FGF: basic fibroblast growth factor
Bio‐Plex: Bio‐Plex Pro multiplex immunoassay system
BMI: body mass index
CCL5: C‐C motif chemokine ligand 5
CD4: cluster of differentiation 4
CD8: cluster of differentiation 8
cDNA: complementary DNA
COVID‐19: coronavirus disease 2019
CV: coefficient of variation
DESeq2: differential expression analysis for sequence count data 2 (R package)
DNA: deoxyribonucleic acid
FOXA1: forkhead box A1
FOXM1: forkhead box M1
FoxO: forkhead box O
G‐CSF: granulocyte colony‐stimulating factor
GM‐CSF: granulocyte–macrophage colony‐stimulating factor
GRCh38: Genome Reference Consortium human reference genome build 38
IFN‐α: interferon alpha
IFN‐γ: interferon gamma
IL‐1: interleukin‐1
IL‐10: interleukin‐10
IL‐12: interleukin‐12
IL‐13: interleukin‐13
IL‐15: interleukin‐15
IL‐17A: interleukin‐17A
IL‐1ra: interleukin‐1 receptor antagonist
IL‐1β: interleukin‐1 beta
IL‐2: interleukin‐2
IL‐4: interleukin‐4
IL‐5: interleukin‐5
IL‐6: interleukin‐6
IL‐7: interleukin‐7
IL‐8: interleukin‐8
IL‐9: interleukin‐9
IP‐10: interferon‐γ–inducible protein 10
IRB: institutional review board
IUGR: intrauterine growth restriction
KEGG: Kyoto Encyclopedia of Genes and Genomes
MAPK: mitogen‐activated protein kinase
MCP‐1: monocyte chemoattractant protein‐1
MIP‐1α: macrophage inflammatory protein‐1 alpha
MIP‐1β: macrophage inflammatory protein‐1 beta
miRBase: microRNA sequence database
miRNA: microRNA
miRNAs: microRNAs
miRTarBase: experimentally validated microRNA–target interaction database
MMP‐2: matrix metalloproteinase‐2
mRNA: messenger RNA
MTI: microRNA–target interaction
NGS: next‐generation sequencing
PBS: phosphate‐buffered saline
PCA: principal component analysis
PDGF‐BB: platelet‐derived growth factor‐BB
PE: preeclampsia
PI3K‐Akt: phosphatidylinositol 3‐kinase–Akt signaling pathway
QIAseq: QIAseq miRNA Library Kit (QIAGEN)
RANTES: regulated on activation, normal T‐cell expressed and secreted
RBC: red blood cell
RBD: receptor binding domain
RIPA: radioimmunoprecipitation assay
RNA: ribonucleic acid
SARS‐CoV‐2: severe acute respiratory syndrome coronavirus 2
TNF‐α: tumor necrosis factor‐alpha
UMI: unique molecular index
UMI‐tools: unique molecular identifier tools
VEGF: vascular endothelial growth factor

## 1 Introduction

COVID‐19, caused by severe acute respiratory syndrome coronavirus 2 (SARS‐CoV‐2), emerged in Wuhan, China, in late 2019 and quickly escalated into a global pandemic, significantly impacting economies and public health. Pregnant women infected with COVID‐19 face increased risks of pregnancy complications, including premature births occurring at rates between 10% and 25% [1]. Protecting the health of mothers and fetuses remains a critical goal for obstetric researchers. As the pandemic progresses, SARS‐CoV‐2 continues to evolve, transitioning from the initial Wuhan strain to subsequent variants such as Alpha, Delta, and currently the Omicron variant, each demonstrating enhanced immune evasion capabilities [2].

MicroRNAs play vital roles in antiviral cellular responses, influencing viral replication and transmission [3, 4]. SARS‐CoV‐2 infection mechanisms likely involve interactions between viral microRNAs and host gene regulatory regions, impacting viral entry, replication, and membrane fusion. Interestingly, human cells produce their own microRNAs as a defense mechanism against viral infection.

Current findings indicate antibody levels against the spike protein and receptor binding domain (RBD) moderately decrease within 8 months post‐infection. Conversely, memory B cells rise between 1 and 8 months after infection, and initial half‐lives of CD8+ and CD4+ T cells range from 3 to 5 months [5]. Cytokine studies have highlighted symptom severity associated with cytokines such as IFN‐α, IP‐10, IFN‐γ, and IL‐6 [6]. COVID‐19 patients typically exhibit elevated cytokines like IFN‐γ, IL‐1, IL‐6, and IL‐12 for at least 2 weeks post‐infection, whereas TNF‐α, IL‐10, IL‐2, and IL‐4 levels remain relatively unchanged [7].

This study aims to evaluate how COVID‐19 affects microRNA expression in placental and amniotic membranes and to determine if microRNA changes correlate with cytokine concentrations. Identifying these biomarkers through placental and amniotic assessments could facilitate early interventions for pregnancy complications, ultimately ensuring maternal‐fetal safety and reducing late‐stage pregnancy risks.

## 2 Materials and Methods

**FIGURE 1:**
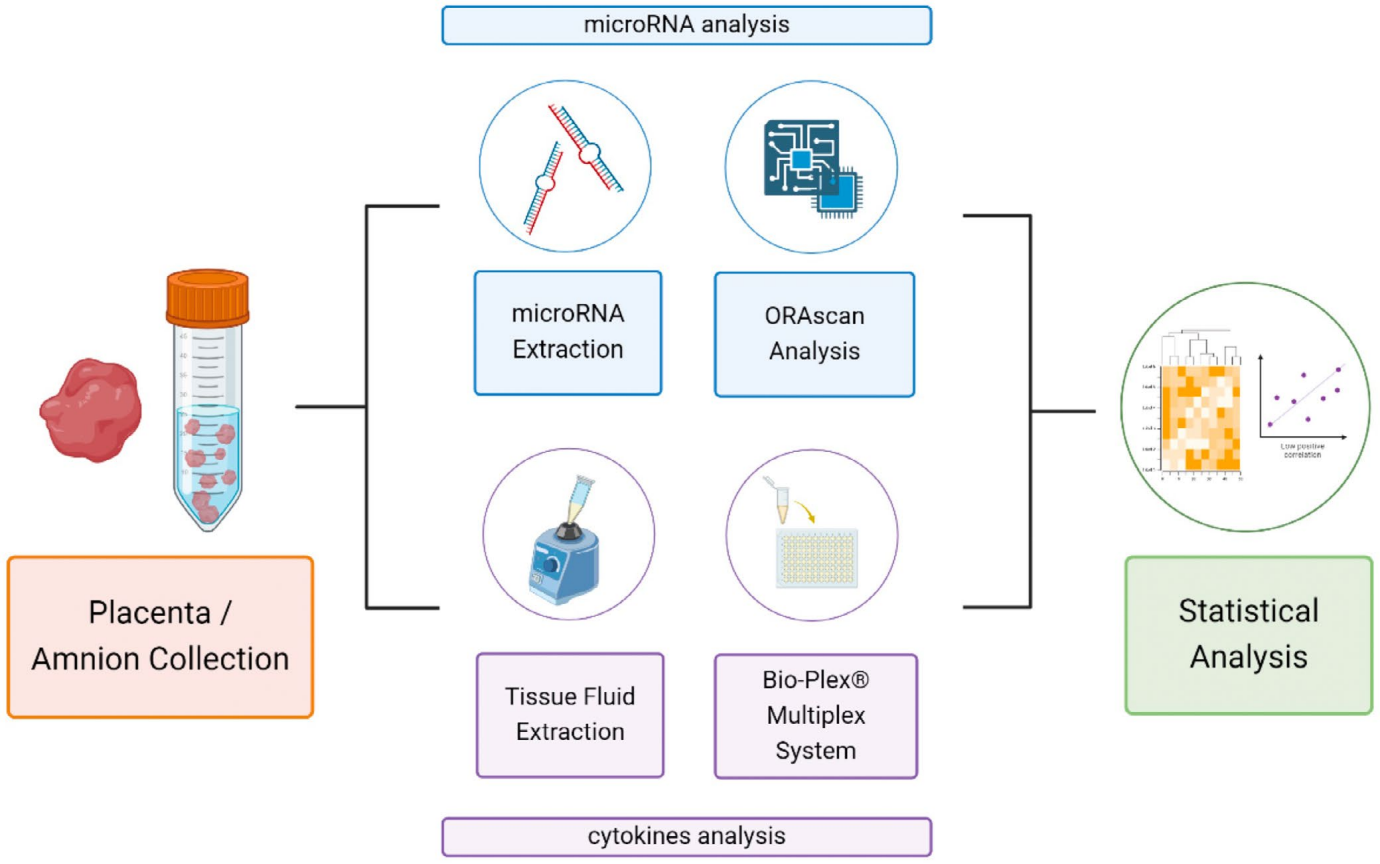
Illustrates the experimental design of our pilot study (created using BioRender). The placenta and amniotic membrane were obtained from pregnant women infected with COVID‐19 and from healthy pregnant women, respectively, and these samples were used for microRNA analysis and cytokines array. Analysis of microRNA and cytokines is used to identify the differences in microRNA and cytokine expression between confirmed cases and non‐cases.

This study collected placental and amniotic membrane samples from 15 pregnant women: 7 infected with COVID‐19 during pregnancy without prior systemic diseases, and 8 healthy controls. Exclusion criteria were: (1) inability to collect samples due to medical emergencies (preterm birth, placental abruption, fetal distress), and (2) unsuitability deemed by the attending physician. All procedures were approved by the institutional review board (IRB number: KMUHIRB‐SV(II)‐20220094). Clinical data, including gestational age at diagnosis, were analyzed alongside samples. The study design is presented in Figure 1.

### 2.1 Extracting Tissue Lysates From Tissue Samples

Cytokine array experiments required extraction of tissue lysates from collected solid tissue blocks. Tissues were minced finely on ice and soaked in 1× RBC Lysis Buffer for 3 min to remove red blood cells, followed by centrifugation at 4000 rpm for 5 min at 4°C. The process was repeated until supernatants were clear. Tissues were then soaked in PBS for 3 min and centrifuged again at the same conditions, discarding supernatants.

Next, 10× RIPA buffer was diluted to 1×, and Protease Inhibitor was added at a 1:200 ratio. Prepared RIPA solution was combined with tissue at a 1:1 volume ratio. The tissue was sonicated on ice in 10‐s intervals until frothy, then incubated on ice for 15 min and centrifuged at 4000 rpm for 30 min at 4°C. The resulting supernatant was used as the tissue lysate for cytokine array analysis.

### 2.2 ORAscan miRNA Library Construction and Sequencing

The miRNA sequencing library was prepared using the QIAseq miRNA Library Kit (QIAGEN, Germany). Steps included: (1) ligation of a pre‐adenylated 3′‐adaptor; (2) ligation of a 5′‐adaptor with sequencing primers; (3) cDNA synthesis with reverse transcription primers containing a unique molecular index (UMI); (4) cDNA cleanup; (5) PCR amplification with barcode primers; and (6) library cleanup. Library quality was assessed using the 5200 Fragment Analyzer System (Agilent Technologies, USA), confirming sizes of 190–220 bp. This size range is strictly expected for the QIAseq system, as the incorporation of UMIs and extended barcode adapters inherently increases the base construct size compared to traditional small RNA kits. Libraries quantified at > 1 ng/μL by Qubit (Thermo Fisher Scientific, USA) were sequenced on the Illumina NextSeq 550 platform following manufacturer guidelines.

### 2.3 Cytokines Array

Cytokine concentrations were analyzed using the Bio‐Plex ProTM Assay (Bio‐Rad Laboratories, USA). Reagents were thawed, and the system was calibrated using Bio‐Plex software. Standards underwent gradient dilution, were mixed, and incubated on ice for 30 min. Samples were diluted per instructions. The 10× magnetic beads were vortexed, diluted to 1×, protected from light, and 50 μL was added to each well. Wells were washed twice with 100 μL Wash Buffer, and 50 μL of samples and standards were added. Plates were incubated at 850 ± 50 rpm for 30 min in the dark.

The 10× Detection Abs were vortexed, diluted to 1×, and added after three washes. Plates were incubated again under the same conditions. The 100× SA‐PE solution was vortexed, diluted to 1×, added after washing, and incubated for 10 min. Finally, wells were washed, and 125 μL of Assay Buffer was added before reading the data.

### 2.4 Statistics Analysis

Raw Fastq sequencing files underwent cleaning to remove adapters and external sequences. miRNA expression analysis involved several steps: umitools identified and removed duplicate reads and adapters [8]; SPORTS software aligned reads to miRNAs, with read lengths set to 15–55 bp allowing one mismatch [9]; DESeq2 normalized data and identified differentially expressed miRNAs (criteria: Log2 fold change ≥ 0.585 or ≤ −0.585, *p* value < 0.05) using Human genome (GRCh38) and miRBase (v22.1) references [10, 11].

Further analysis assessed sample correlations and biological functions of miRNAs. Unsupervised hierarchical clustering with the R package “pheatmap” visualized correlations between samples and miRNA profiles. Differentially expressed miRNAs underwent microRNA target interaction (MTI) analysis using miRTarBase [12], retaining strongly supported interactions. Gene enrichment analysis utilized the R package clusterProfiler and KEGG pathways [13, 14] focusing on filtered target gene lists.

## 3 Results

In this study, placental and amniotic samples were collected from 15 pregnant women, some diagnosed with COVID‐19 at delivery. This group included seven women who contracted COVID‐19 during pregnancy, with no pre‐existing systemic conditions before infection. The control group comprised eight healthy pregnant women who remained COVID‐19 free. Sample details of these 15 women, including maternal age, parity, gestational age, infection‐delivery interval, BMI, newborn sex, and newborn weight, are presented in Table S1. Among these, five pregnant women were excluded from subsequent analyses, with the detailed exclusion rationale discussed later.

### 3.1 MicroRNA Profile Changes With COVID‐19 Infection

**FIGURE 2:**
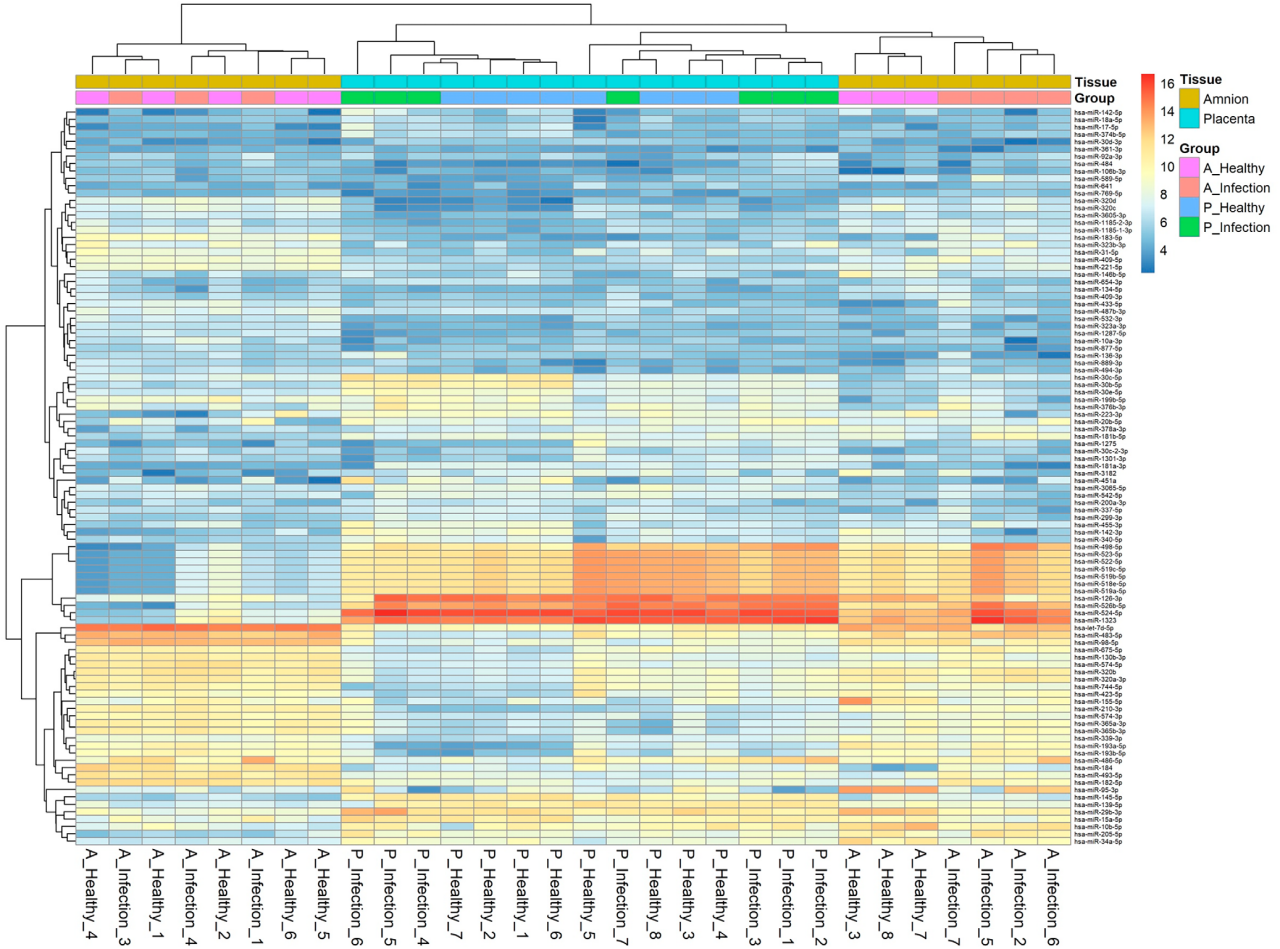
Heatmap of microRNA expression from 30 specimens. Placental samples are labeled as P, and amniotic samples are labeled as A. Seven confirmed samples of placenta and amnion tissue and eight healthy samples of placenta and amnion tissue in total. The heatmap visualizes the relative expression levels of microRNAs through microRNA NGS.

We examined microRNA differential expression in placental and amniotic samples from all 15 women, totaling 30 data sets. Placental samples were labeled as P, amniotic samples as A. Figure 2 illustrates a heatmap of the top 100 microRNAs with the highest coefficient of variation (CV) after next‐generation sequencing (NGS). The heatmap clustering shows distinct gene expression between placenta and amnion samples, while diagnosed versus healthy samples were not clearly separated. This observation suggests that intrinsic biological differences between placental and amniotic tissues are the main source of transcriptomic variance. Because the COVID‐19 cases in our cohort were mild, the infection likely caused subtle molecular changes rather than global transcriptomic alterations capable of overriding tissue‐specific differences.

Prior to detailed analysis, an exploratory outlier identification step was performed to manage the profound inter‐individual biological heterogeneity frequently observed in human placental and amniotic tissues. Outliers were identified among four groups (placenta, amnion, diagnosed, healthy) to ensure strict data quality and prevent false‐positive signals in our small cohort. Sample P_infection_6 emerged as a significant outlier in both placental and diagnosed groups based on the frequency of microRNA outlier counts, and was subsequently excluded from further analysis (Figure S1). Principal component analysis (PCA) on cytokine data sets from all 30 samples identified four outliers: two from amniotic tissue (A_healthy_7, A_healthy_6) and two from placental tissue (P_healthy_8, P_infection_7), shown in Figure S2. Three of these outliers belonged to healthy women and one to a diagnosed woman. To maintain a perfectly paired study design for matched placenta‐amnion comparisons, the corresponding samples from these outliers (Healthy_6, Healthy_7, Healthy_8, Infection_6, Infection_7) were also excluded. Thus, the final analysis included 10 women (5 diagnosed, 5 healthy). We acknowledge that relying on PCA and outlier counts for sample exclusion, in the absence of overt technical preparation failures, is a highly stringent approach that risks removing genuine biological variability. This step was taken strictly to isolate a homogenous core molecular signature, albeit at the cost of statistical power.

### 3.2 MicroRNA Associated With COVID‐19 Infection in Placental Tissue and Amnion Membrane

**FIGURE 3:**
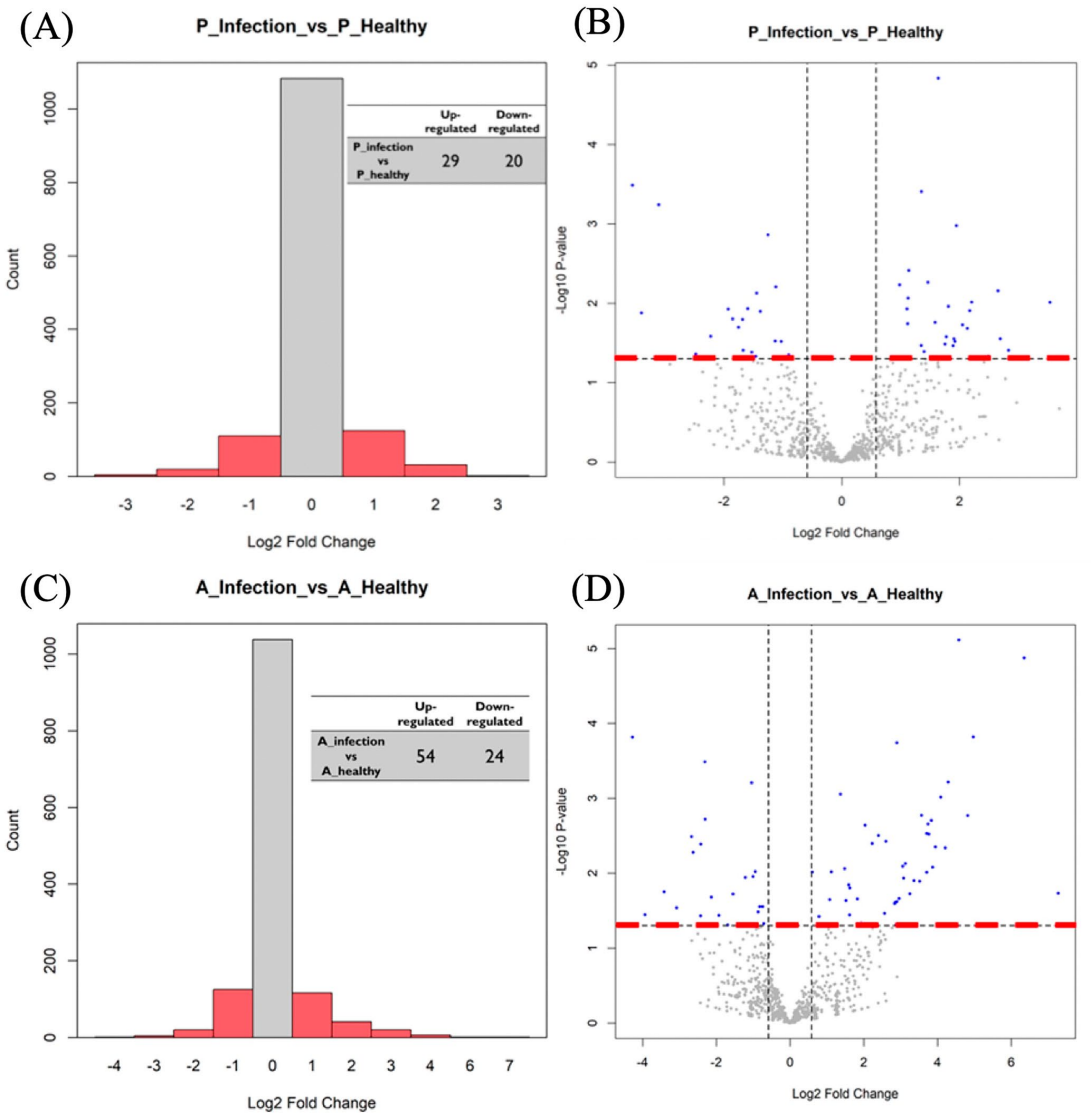
(A) Histogram and (B) volcano plot of placenta samples (*n* = 10), and (C) histogram and (D) volcano plot of amnion samples (*n* = 10). These pictures show the differences in microRNA expression between samples from five confirmed pregnant women and five healthy pregnant women. The *X*‐axis in a histogram shows positive values indicating upregulation of microRNA after diagnosis, and the *Y*‐axis represents the number of microRNAs. The volcano plot indicates microRNAs with significant differences. The blue dots above the red dashed line represent microRNAs with significant differences.

Histograms and volcano plots analyzed placental microRNA data (five COVID‐19, five healthy) to confirm data reliability (Figure 3A,B). Histograms indicated most microRNAs had similar expression in both groups, with 29 upregulated and 20 downregulated microRNAs identified. Volcano plots presented statistically significant microRNAs (blue dots above red dashed line) with clear trends in upregulation and downregulation.

Using KEGG database resources, we identified the top 10 pathways related to placental microRNAs (Table S2), involving cellular aging, proliferation, and viral infection. Amniotic membrane microRNA data were similarly analyzed via histograms and volcano plots (Figure 3C,D), revealing a substantial upregulation of genes (54 upregulated, 24 downregulated). Increased microRNA upregulation suggests reduced mRNA degradation or cytokine concentration. The volcano plots confirmed significantly upregulated microRNAs.

The top 10 pathways involving differentially expressed amniotic microRNAs are listed in Table S3. A combined analysis identified intersections of differentially expressed microRNAs in both tissues, finding four shared microRNAs: hsa‐miR‐20a‐5p, hsa‐miR‐20b‐5p, hsa‐miR‐25‐3p, and hsa‐miR‐362‐5p (Table 1). Three microRNAs showed consistent upward trends in both tissues post‐infection, while one (miR‐362‐5p) had opposite expression trends—upregulated in placenta but downregulated in amnion.

**TABLE 1:** Expression levels of microRNAs with differential expression in both placenta and amnion.

	Placenta		Amnion	
	Log2FC	*p*	Log2FC	*p*
hsa‐miR‐20a‐5p	1.64	1.47 × 10^−5^	0.79	0.0377
hsa‐miR‐20b‐5p	1.59	0.0174	1.62	0.0158
hsa‐miR‐25‐3p	1.13	0.0086	1.13	0.0096
hsa‐miR‐362‐5p	0.98	0.0059	−0.71	0.0469

*Note:* Take the fold change of each microRNA detected by microRNA NGS and convert it to log2 for comparison.

Subsequently, pathway analysis for the three consistent microRNAs was conducted (Table S4). Cellular aging, PI3K‐Akt, FoxO, and MAPK signaling pathways were frequently highlighted, underscoring COVID‐19's influence on microRNA regulation in placental and amniotic tissues.

### 3.3 Analysis of Cytokine Changes With or Without COVID‐19 Infection

Cytokine analysis validated microRNA findings by measuring 27 cytokine concentrations in placental and amniotic samples. No significant cytokine differences emerged between diagnosed and healthy groups in either tissue type. Correlation analyses between cytokines and microRNAs were thus performed.

**FIGURE 4:**
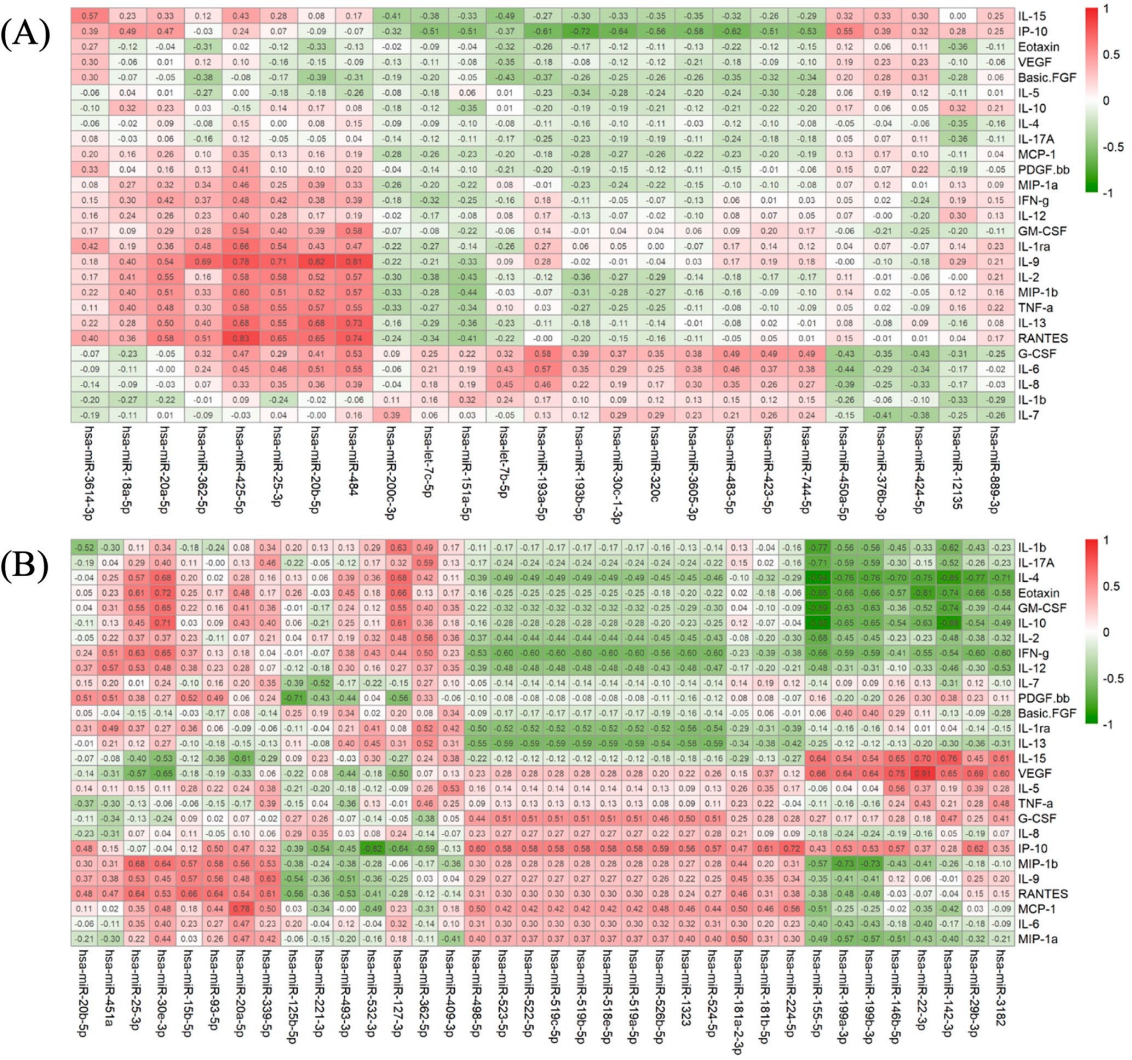
Correlation analysis of (A) placental and (B) amniotic microRNAs with 27 cytokines. The 27 cytokines tested are IL‐1β, IL‐1ra, IL‐2, IL‐4, IL‐5, IL‐6, IL‐7, IL‐8, IL‐9, IL‐10, IL‐12, IL‐13, IL‐15, IL‐17A, basic‐FGF, Eotaxin, G‐CSF, GM‐CSF, IFN‐γ, IP‐10, MCP‐1, MIP‐1α, MIP‐1β, PDGF‐BB, RANTES, TNF‐α, and VEGF. In the diagram, red indicates a positive correlation, while green indicates a negative correlation. In placenta sample, IL‐9 and RANTES have strong correlation with some microRNAs; In amnion sample, five cytokines have strong correlation with some microRNAs, which are IL‐4, IL‐10, Eotaxin, GM‐CSF, VEGF and IP‐10.

Figure 4A demonstrates correlations between placental microRNAs and cytokines, highlighting three significantly correlated pairs (|*r*| > 0.8), summarized in Table 2A. MiR‐20b‐5p and miR‐484 positively correlated with increased IL‐9 concentrations. IL‐9 regulates immune responses, inflammation, and allergic reactions, showing elevated levels after COVID‐19 infection in prior studies [15]. Despite nonsignificant differences in overall IL‐9 levels between groups, the significant positive correlation indicates IL‐9 upregulation after infection, likely obscured due to mild cases.

**TABLE 2:** Significantly correlated microRNAs and cytokines in (A) placenta and (B) amnion, *n* = 10.

(A)			
MicroRNA	Cytokine	*r*	*p*
hsa‐miR‐20b‐5p	IL‐9	0.82	0.0036
hsa‐miR‐484	IL‐9	0.81	0.0049
hsa‐miR‐425‐5p	RANTES	0.83	0.0027

(B)			
MicroRNA	Cytokine	*r*	*p*
hsa‐miR‐142‐3p	IL‐4	−0.85	0.0017
hsa‐miR‐142‐3p	IL‐10	−0.88	0.0007
hsa‐miR‐155‐5p	Eotaxin	−0.86	0.0013
hsa‐miR‐155‐5p	GM‐CSF	−0.89	0.0006
hsa‐miR‐155‐5p	IL‐4	−0.94	0.0000
hsa‐miR‐155‐5p	IL‐10	−0.95	0.0000
hsa‐miR‐22‐3p	Eotaxin	−0.81	0.0049
hsa‐miR‐22‐3p	VEGF	0.91	0.0002
hsa‐miR‐532‐3p	IP‐10	−0.82	0.0034

*Note:* There are five confirmed cases and five healthy cases.

Another notable positive correlation was observed between microRNAs and RANTES (CCL5). RANTES plays crucial roles in inflammatory immune responses and neurodegeneration, potentially contributing to neurological COVID‐19 symptoms like brain fog [16]. While direct literature linking RANTES to COVID‐19‐induced neurological effects is limited, the correlation highlights important immune‐regulatory mechanisms activated during infection.

Figure 4B demonstrated the correlation analyses of amniotic cytokines and microRNAs, and nine significantly correlated pairs were identified (|*r*| > 0.8; Table 2B). Four microRNAs (miR‐142‐3p, miR‐155‐5p, miR‐22‐3p, miR‐532‐3p) correlated strongly with cytokine concentrations, mostly demonstrating inhibitory roles in cytokine expression.

Specifically, miR‐142‐3p and miR‐155‐5p inversely correlated with IL‐4 and IL‐10 concentrations; cytokine levels rose when microRNA expression decreased. Additionally, miR‐155‐5p upregulation suppressed Eotaxin and GM‐CSF. MiR‐22‐3p showed an inverse correlation with Eotaxin but a positive correlation with VEGF. MiR‐532‐3p correlated strongly with IP‐10 concentration regulation. Although IP‐10 levels showed no significant differences between groups, the observed correlation suggests COVID‐19‐induced microRNA alterations might influence IP‐10 regulation indirectly.

These cytokine‐microRNA correlations illustrate a sophisticated regulatory network influenced by COVID‐19 infection. Placental tissue displayed microRNAs enhancing inflammatory cytokines like IL‐9 and RANTES, potentially affecting immune and neurological functions. Conversely, amniotic membranes exhibited predominantly inhibitory microRNA‐cytokine relationships, suggesting modulation of immune response and cytokine production in fetal tissues following maternal infection.

Overall, this study confirms specific and targeted alterations in microRNA expression profiles in placental and amniotic tissues due to COVID‐19. These alterations notably influence cytokine regulation, affecting pathways integral to immune response, inflammation, cellular aging, and proliferation. Understanding these molecular changes provides critical insights into COVID‐19's impacts on pregnancy, maternal‐fetal interactions, and potential implications for fetal development and long‐term health outcomes.

## 4 Discussion

The microRNA expression patterns in the placenta and amnion membranes are consistent across samples. Notably, three microRNAs exhibit similar regulatory mechanisms after COVID‐19 infection. According to the literature, SARS‐CoV‐2 relies on its own microRNAs to interact with regulatory regions, influencing viral replication, membrane fusion, and cell entry [3]. These interactions trigger a cascade of signals that reshape the human immune response to the viral invasion. Our combined analysis of both tissues reveals a compartmentalized maternal‐fetal immune dynamic: while the placenta acts as an active inflammatory barrier against viral invasion, the amniotic membrane serves as an immunomodulatory buffer. Their divergent microRNA‐cytokine regulatory patterns reflect a coordinated effort to protect the fetus from excessive maternal inflammation.

The differentially expressed microRNAs in both placental and amniotic samples, miR‐20a‐5p and miR‐20b‐5p, have been previously implicated in COVID‐19 severity and pregnancy complications [17, 18], particularly preeclampsia (PE) [19]. Studies on plasma samples from COVID‐19 patients have shown that these microRNAs can predict disease severity, mortality rates, and prognostic outcomes [18], highlighting their potential as biomarkers. In the context of pregnancy, Wang et al. and Jin et al. demonstrated that miR‐20a and miR‐20b inhibit trophoblast function by targeting FOXA1 and MMP‐2, respectively, affecting cell proliferation, invasion, and differentiation [20].

Although our study focused on mild COVID‐19 cases, the observed upregulation of miR‐20a‐5p and miR‐20b‐5p in placental and amniotic samples aligns with these previous findings. As shown in our unsupervised clustering analysis, global microRNA profiles did not clearly separate infected and healthy groups, mainly because of tissue‐specific variance. This suggests that mild COVID‐19 infection may induce subtle but specific alterations in placental function, potentially affecting placental development and pregnancy outcomes. The identification of these microRNA signatures despite strong tissue‐type variance highlights their sensitivity and potential value as early indicators of viral effects in mild cases. While we did not include severe cases, comparing immune mechanisms between mild cases and healthy pregnant women showed similar phenomena to those observed in more severe COVID‐19 cases and PE.

These findings underscore the importance of further research to fully elucidate the long‐term implications of these changes on maternal and fetal health. Additionally, they suggest that microRNA testing could be valuable for monitoring post‐diagnosis outcomes and the health status of pregnant women and their fetuses. Future studies should aim to validate these results in larger cohorts, including severe cases, and explore the potential of using these microRNAs as diagnostic tools and targets for therapeutic interventions in both COVID‐19 and pregnancy complications.

miR‐25‐3p has emerged as a significant microRNA in both COVID‐19 diagnosis and pregnancy complications. It has been used to distinguish COVID‐19 from other pneumonias and is associated with cytokine IL‐10 dysregulation after SARS‐CoV‐2 infection [21]. In pregnancy, miR‐25‐3p plays a crucial role in PE and intrauterine growth restriction (IUGR) [22, 23]. Its upregulation in the placenta during PE and in maternal circulation during IUGR suggests its involvement in trophoblast function, invasion, and fetal growth restriction. Although our study didn't specifically include fetal growth restriction cases, the impact of COVID‐19 on placental and amnion membranes, coupled with miR‐25‐3p's known roles, remains significant. These findings highlight miR‐25‐3p's potential as a biomarker for early diagnosis of both COVID‐19 and pregnancy complications and as a possible therapeutic target.

miR‐142‐3p and miR‐155 demonstrate complex, context‐dependent roles in pregnancy complications and COVID‐19 responses. In PE, both are typically upregulated in placental tissue, with miR‐142‐3p inhibiting trophoblast proliferation and invasion via FOXM1 regulation, and miR‐155 suppressing extravillous trophoblast cell proliferation and migration by downregulating cyclin D1 [19]. Both microRNAs show elevated expression in exosomes of gestational diabetes mellitus patients. Our study of mild COVID‐19 cases revealed unexpected findings: miR‐142‐3p was downregulated in amniotic samples, aligning with observations from blood samples in other COVID‐19 studies [24]. This downregulation potentially increases IL‐4 and IL‐10 levels [25]. Intriguingly, miR‐155‐5p, normally highly expressed in severe COVID‐19 [26], was downregulated in our amniotic samples. Our amniotic sample analysis showed that miR‐142‐3p and miR‐155‐5p suppress IL‐4 and IL‐10 concentrations, indicating an inverse correlation between these microRNAs and cytokine levels. Despite these molecular changes, we observed no significant cytokine differences between diagnosed and healthy groups, possibly due to the mild nature of our cases. These findings highlight the intricate roles of miR‐142‐3p and miR‐155 in trophoblast function, inflammatory responses, and immune regulation. Importantly, the relationships identified between microRNAs and cytokines in this study are based entirely on correlation analyses. We acknowledge that correlation does not establish causality, and therefore the proposed immunoregulatory networks remain speculative. Future functional validations, such as in vitro cell line experiments or in vivo models, are necessary to confirm whether these microRNAs mechanistically drive the observed cytokine alterations.

This study provides novel insights into the effects of mild COVID‐19 on microRNA expression in placental and amniotic tissues of pregnant women, particularly focusing on miR‐142‐3p, miR‐155, and miR‐25‐3p. Our findings corroborate previously reported SARS‐CoV‐2 infection mechanisms and offer new perspectives on the complex, context‐dependent roles of these microRNAs in pregnancy complications and viral responses. However, we must acknowledge several major limitations of our study. First, the sample size is extremely small, with the final analysis including only 10 subjects (5 COVID‐19 and 5 healthy). This reduction from the initial cohort was a necessary quality‐control measure to exclude outliers and prevent false‐positive artifacts, though it inherently limits our statistical power. Second, our findings are based solely on NGS and Bio‐Plex arrays without subsequent independent validation via quantitative PCR (qPCR) or an external validation cohort. Despite the limited sample size and the inclusion of only mild cases, our results provide an exploratory basis for using microRNAs and cytokines to assess placental conditions post‐COVID‐19 infection. The unexpected downregulation of miR‐142‐3p and miR‐155‐5p in our samples, contrasting with their typical upregulation in PE and severe COVID‐19, underscores the need for further research to fully elucidate their functions in various contexts. This pilot study paves the way for developing new diagnostic and monitoring tools that could be crucial in prenatal care. Future research must expand the sample size, include cases of varying severity, conduct long‐term follow‐ups, and validate these biomarkers in independent cohorts and blood samples. By integrating microRNA analysis into routine prenatal screenings, we may develop more precise risk assessment tools, offering better guidance for pregnant women's care during and after the COVID‐19 pandemic. Ultimately, this research advances our understanding of how COVID‐19 affects pregnancy and provides new directions for future prenatal care practices.

This study provides novel insights into the effects of mild COVID‐19 on microRNA expression in placental and amniotic tissues of pregnant women, particularly focusing on miR‐142‐3p, miR‐155, and miR‐25‐3p. Our findings corroborate previously reported SARS‐CoV‐2 infection mechanisms and offer new perspectives on the complex, context‐dependent roles of these microRNAs in pregnancy complications and viral responses. Despite the limited sample size and the inclusion of only mild cases, our results provide a reliable basis for using microRNAs and cytokines to assess placental conditions post‐COVID‐19 infection. Furthermore, a major limitation of our study is the absence of data linking these molecular changes to clinically meaningful endpoints. Because our cohort consisted exclusively of mild cases with favorable pregnancy outcomes, we lacked the clinical variance necessary to directly correlate these microRNA alterations with disease severity or long‐term neonatal health. The unexpected downregulation of miR‐142‐3p and miR‐155‐5p in our samples, contrasting with their typical upregulation in PE and severe COVID‐19, underscores further research to fully elucidate their functions in various contexts. This study paves the way for developing new diagnostic and monitoring tools that could be crucial in prenatal care. Future research should expand the sample size, include cases of varying severity, conduct long‐term follow‐ups, and validate these biomarkers in blood samples. By integrating microRNA analysis into routine prenatal screenings, we may develop more precise risk assessment tools, offering better guidance for pregnant women's care during and after the COVID‐19 pandemic. Ultimately, this research advances our understanding of how COVID‐19 affects pregnancy and provides new directions for future prenatal care practices. Additionally, the decision to exclude one‐third of our cohort (5 out of 15 subjects) based primarily on exploratory PCoA and outlier count visualizations introduces a distinct risk of selection bias. While intended to reduce extreme inter‐individual heterogeneity, this filtering step may have discarded biologically meaningful variability, further emphasizing the need for our findings to be validated in larger, unfiltered cohorts.

## 5 Conclusions

We utilized microRNA NGS system and cytokine array analysis to examine the microRNA and cytokine profiles of placental and amniotic samples from 10 pregnant women. Our aim was to investigate the immunogenicity of the placenta following COVID‐19 infection and compare whether the placenta and the inner amniotic membrane share common immune mechanisms. It was observed that three microRNAs, hsa‐miR‐20a‐5p, hsa‐miR‐20b‐5p, and hsa‐miR‐25‐3p, exhibited similar regulatory trends in both placental and amniotic tissues. These microRNAs have also been confirmed as biomarkers for COVID‐19 and are related to fetal development.

Additionally, we compared cytokine profiles between samples from 5 COVID‐19 positive pregnant women and 5 healthy pregnant women, finding no significant differences. However, we discovered a significant correlation between the expression of seven microRNAs and the corresponding cytokine concentrations, indicating that microRNA regulation can affect cytokine levels, either increasing or decreasing them. Relevant cytokines, such as IL‐4, IL‐9, IL‐10, RANTES, and IP‐10, were identified in the literature as being associated with COVID‐19. This suggests that microRNAs are feasible biomarkers for assessing COVID‐19 infection. In the future, incorporating microRNA detection into prenatal and postnatal care could help ensure the safety of both mother and child after SARS‐CoV‐2 infection.

## Funding

This research was funded by Taiwan's Chang Gung Medical Foundation (grant number CRRPG2L0011, CMRPG2L0261, and CMRPVVM0121), Taiwan's National Science and Technology Council (grant number 112‐2622‐E‐007‐028), and Taiwan's Kaohsiung Medical University Hospital (grant number KMUH113‐3M27 and KMUH114‐4M41).

## Ethics Statement

The study was conducted in accordance with the guidelines of the Declaration of Helsinki and was approved by the Institutional Review Board (IRB) of Kaohsiung Medical University Hospital (IRB No. KMUHIRB‐SV(II)‐20,210,087) on August 7, 2021.

## Conflicts of Interest

The authors declare no conflicts of interest.

## Supporting information


**Data S1:** Supporting Information.
**Figure S1:** Distribution chart of microRNA outliers. This chart illustrates the number of times each sample is determined to be an outlier for various microRNAs based on the four combinations of placenta, amnion, diagnosed, and healthy. P_infection_6 which is highlighted in red boxes was identified as a significant outlier in both the placental and diagnosed groups.
**Figure S2:** Principal component analysis (PCA) plot of cytokines level from 15 pregnant women. Seven confirmed samples of placenta and amnion tissue and eight healthy samples of placenta and amnion tissue in total. The red boxes in the figure indicate the outlier samples. Among the four outlier samples, two are from amniotic tissue and two from placental tissue.
**Table S1:** Participant demographic and clinical data.
**Table S2:** Top 10 KEGG Pathways for microRNA with differential expression in placental tissue.
**Table S3:** Top 10 KEGG Pathways for microRNA with differential expression in amnion membrane.
**Table S4:** Top 10 KEGG Pathways for intersecting microRNAs with differential expression in placenta and amnion.

## Data Availability

The data that support the findings of this study are available on request from the corresponding author. The data are not publicly available due to privacy or ethical restrictions.
