# Supplementary material for: Alterations in MicroRNA and Cytokine Expressions in Placental and Amniotic Tissues of COVID‐19 Affected Pregnant Women
Source: Kaohsiung J Med Sci. 2026 Apr 2;42(11):e70207. doi: 10.1002/kjm2.70207 (PMC13399688; doi:10.1002/kjm2.70207)
Supplement: Supplementary file 1 [file KJM2-42-e70207-s001.zip › Supplementray table.docx]

**Table S1.** Participant demographic and clinical data.

|  | **Age** | **Parity** | **Weeks of gestation at delivery** | **COVID-19 vaccination** | **interval between the infection of COVID-19 and the collection of sample (day of delivery) (weeks)** | **BMI** | **sex of newborn** | Weight of newborn (g) |
| --- | --- | --- | --- | --- | --- | --- | --- | --- |
| Infection_1 | 32 | 2 | 39 | Moderna/ Moderna/ Moderna/ Moderna Bivalent vaccine | 27 | 24.44 | M | 3180 |
| Infection_2 | 41 | 1 | 39 | AZ/ AZ/ Moderna/ Moderna Bivalent vaccine | 14 | 22.18 | M | 2470 |
| Infection_3 | 32 | 1 | 38 | AZ/ AZ/ Moderna | 30 | 31.63 | M | 3535 |
| Infection_4 | 34 | 2 | 39 | AZ/ AZ/ Moderna/ Moderna | 12 | 29.48 | M | 3030 |
| Infection_5 | 25 | 0 | 40 | Moderna/ Moderna/ Moderna | 27 | 22.72 | F | 3095 |
| Infection_6* | 28 | 1 | 39 | AZ/ AZ/ Moderna | 18 | 31.50 | F | 2920 |
| Infection_7* | 32 | 1 | 37 | AZ/ Moderna/ Moderna/ Moderna Bivalent vaccine | 25 | 26.84 | F | 2815 |
| Healthy_1 | 37 | 3 | 40 | Moderna/ Moderna/ Moderna | - | 25.52 | M | 3085 |
| Healthy_2 | 39 | 2 | 38 | Moderna/ Moderna/ Moderna/ Moderna Bivalent vaccine | - | 26.30 | M | 2965 |
| Healthy_3 | 42 | 2 | 39 | Moderna/ Moderna/ Moderna/ Moderna | - | 24.52 | F | 3280 |
| Healthy_4 | 33 | 2 | 40 | Moderna/ Moderna/ Moderna/ Moderna Bivalent vaccine | - | 27.51 | F | 3315 |
| Healthy_5 | 29 | 1 | 40 | AZ/ Moderna/ Moderna/ Moderna | - | 28.61 | F | 3470 |
| Healthy_6* | 30 | 1 | 39 | Moderna/ Moderna/ BNT/ Moderna Bivalent vaccine | - | 21.64 | M | 2895 |
| Healthy_7* | 30 | 1 | 39 | AZ/ AZ/ Moderna/ Moderna Bivalent vaccine | - | 24.50 | M | 2850 |
| Healthy_8* | 31 | 1 | 38 | AZ/ AZ/ Moderna/ Moderna | - | 31.98 | F | 3175 |

**Table S2.** Top 10 KEGG Pathways for microRNA with differential expression in placental tissue.

| **ID** | **Description** | **Count** | **P value** | **Q value** |
| --- | --- | --- | --- | --- |
| hsa04218 | Cellular senescence | 42 | 2.28E-27 | 7.28E-26 |
| hsa04151 | PI3K-Akt signaling pathway | 60 | 8.88E-27 | 2.13E-25 |
| hsa05161 | Hepatitis B | 40 | 1.60E-24 | 2.56E-23 |
| hsa04010 | MAPK signaling pathway | 50 | 2.68E-22 | 2.85E-21 |
| hsa01521 | EGFR tyrosine kinase inhibitor resistance | 26 | 1.04E-19 | 6.66E-19 |
| hsa01522 | Endocrine resistance | 28 | 2.86E-19 | 1.52E-18 |
| hsa04933 | AGE-RAGE signaling pathway in diabetic complications | 28 | 5.22E-19 | 2.63E-18 |
| hsa04068 | FoxO signaling pathway | 31 | 1.36E-18 | 6.51E-18 |
| hsa05163 | Human cytomegalovirus infection | 39 | 4.66E-18 | 2.12E-17 |
| hsa05169 | Epstein-Barr virus infection | 36 | 3.92E-17 | 1.71E-16 |

**Table S3.** Top 10 KEGG Pathways for microRNA with differential expression in amnion membrane.

| **ID** | **Description** | **Count** | **P value** | **Q value** |
| --- | --- | --- | --- | --- |
| hsa04151 | PI3K-Akt signaling pathway | 91 | 7.55E-32 | 2.19E-30 |
| hsa04010 | MAPK signaling pathway | 82 | 2.65E-31 | 5.50E-30 |
| hsa04933 | AGE-RAGE signaling pathway in diabetic complications | 48 | 4.11E-31 | 7.47E-30 |
| hsa05161 | Hepatitis B | 60 | 6.18E-31 | 9.98E-30 |
| hsa04068 | FoxO signaling pathway | 52 | 1.28E-28 | 1.55E-27 |
| hsa04668 | TNF signaling pathway | 48 | 7.16E-28 | 7.43E-27 |
| hsa04218 | Cellular senescence | 55 | 3.78E-27 | 3.66E-26 |
| hsa01521 | EGFR tyrosine kinase inhibitor resistance | 39 | 4.85E-26 | 4.15E-25 |
| hsa01522 | Endocrine resistance | 42 | 7.09E-25 | 5.42E-24 |
| hsa05165 | Human papillomavirus infection | 76 | 1.23E-23 | 8.51E-23 |

**Table S4.** Top 10 KEGG Pathways for intersecting microRNAs with differential expression in placenta and amnion.

| **ID** | **Description** | **Count** | **P value** | **Q value** |
| --- | --- | --- | --- | --- |
| hsa04218 | Cellular senescence | 16 | 1.23E-12 | 4.37E-11 |
| hsa05169 | Epstein-Barr virus infection | 16 | 6.57E-11 | 1.49E-09 |
| hsa04151 | PI3K-Akt signaling pathway | 20 | 1.22E-10 | 1.49E-09 |
| hsa01522 | Endocrine resistance | 12 | 1.26E-10 | 1.49E-09 |
| hsa05161 | Hepatitis B | 14 | 3.67E-10 | 3.90E-09 |
| hsa04110 | Cell cycle | 13 | 2.81E-09 | 1.87E-08 |
| hsa05167 | Kaposi sarcoma-associated herpesvirus infection | 13 | 3.65E-08 | 2.04E-07 |
| hsa04068 | FoxO signaling pathway | 11 | 4.46E-08 | 2.37E-07 |
| hsa01524 | Platinum drug resistance | 8 | 4.45E-07 | 1.89E-06 |
| hsa05165 | Human papillomavirus infection | 15 | 5.24E-07 | 2.08E-06 |
