# Supplementary material for: Alterations in MicroRNA and Cytokine Expressions in Placental and Amniotic Tissues of COVID‐19 Affected Pregnant Women
Source: Kaohsiung J Med Sci. 2026 Apr 2:e70207. Online ahead of print. doi: 10.1002/kjm2.70207 (PMC13399688; doi:10.1002/kjm2.70207)
Supplement: Supplementary file 1 — Data S1: Supporting Information. Figure S1: Distribution chart of microRNA outliers. This chart illustrates the number of times each sample is determined to be an outlier for various microRNAs based on the four combinations of placenta, amnion, diagnosed, and healthy. P_infection_6 which is highlighted in red boxes was identified as a significant outlier in both the placental and diagnosed groups. Figure S2: Principal component analysis (PCA) plot of cytokines level from 15 pregnant women. Seven confirmed samples of placenta and amnion tissue and eight healthy samples of placenta and amnion tissue in total. The red boxes in the figure indicate the outlier samples. Among the four outlier samples, two are from amniotic tissue and two from placental tissue. Table S1: Participant demographic and clinical data. Table S2: Top 10 KEGG Pathways for microRNA with differential expression in placental tissue. Table S3: Top 10 KEGG Pathways for microRNA with differential expression in amnion membrane. Table S4: Top 10 KEGG Pathways for intersecting microRNAs with differential expression in placenta and amnion. [file KJM2-9999-e70207-s001.zip › Supplementray table.docx]

**Table S1.** Participant demographic and clinical data.

|  | **Age** | **Parity** | **Weeks of gestation at delivery** | **COVID-19 vaccination** | **interval between the infection of COVID-19 and the collection of sample (day of delivery) (weeks)** | **BMI** | **sex of newborn** | Weight of newborn (g) |
| --- | --- | --- | --- | --- | --- | --- | --- | --- |
| Infection_1 | 32 | 2 | 39 | Moderna/ Moderna/ Moderna/ Moderna Bivalent vaccine | 27 | 24.44 | M | 3180 |
| Infection_2 | 41 | 1 | 39 | AZ/ AZ/ Moderna/ Moderna Bivalent vaccine | 14 | 22.18 | M | 2470 |
| Infection_3 | 32 | 1 | 38 | AZ/ AZ/ Moderna | 30 | 31.63 | M | 3535 |
| Infection_4 | 34 | 2 | 39 | AZ/ AZ/ Moderna/ Moderna | 12 | 29.48 | M | 3030 |
| Infection_5 | 25 | 0 | 40 | Moderna/ Moderna/ Moderna | 27 | 22.72 | F | 3095 |
| Infection_6* | 28 | 1 | 39 | AZ/ AZ/ Moderna | 18 | 31.50 | F | 2920 |
| Infection_7* | 32 | 1 | 37 | AZ/ Moderna/ Moderna/ Moderna Bivalent vaccine | 25 | 26.84 | F | 2815 |
| Healthy_1 | 37 | 3 | 40 | Moderna/ Moderna/ Moderna | - | 25.52 | M | 3085 |
| Healthy_2 | 39 | 2 | 38 | Moderna/ Moderna/ Moderna/ Moderna Bivalent vaccine | - | 26.30 | M | 2965 |
| Healthy_3 | 42 | 2 | 39 | Moderna/ Moderna/ Moderna/ Moderna | - | 24.52 | F | 3280 |
| Healthy_4 | 33 | 2 | 40 | Moderna/ Moderna/ Moderna/ Moderna Bivalent vaccine | - | 27.51 | F | 3315 |
| Healthy_5 | 29 | 1 | 40 | AZ/ Moderna/ Moderna/ Moderna | - | 28.61 | F | 3470 |
| Healthy_6* | 30 | 1 | 39 | Moderna/ Moderna/ BNT/ Moderna Bivalent vaccine | - | 21.64 | M | 2895 |
| Healthy_7* | 30 | 1 | 39 | AZ/ AZ/ Moderna/ Moderna Bivalent vaccine | - | 24.50 | M | 2850 |
| Healthy_8* | 31 | 1 | 38 | AZ/ AZ/ Moderna/ Moderna | - | 31.98 | F | 3175 |

**Table S2.** Top 10 KEGG Pathways for microRNA with differential expression in placental tissue.

| **ID** | **Description** | **Count** | **P value** | **Q value** |
| --- | --- | --- | --- | --- |
| hsa04218 | Cellular senescence | 42 | 2.28E-27 | 7.28E-26 |
| hsa04151 | PI3K-Akt signaling pathway | 60 | 8.88E-27 | 2.13E-25 |
| hsa05161 | Hepatitis B | 40 | 1.60E-24 | 2.56E-23 |
| hsa04010 | MAPK signaling pathway | 50 | 2.68E-22 | 2.85E-21 |
| hsa01521 | EGFR tyrosine kinase inhibitor resistance | 26 | 1.04E-19 | 6.66E-19 |
| hsa01522 | Endocrine resistance | 28 | 2.86E-19 | 1.52E-18 |
| hsa04933 | AGE-RAGE signaling pathway in diabetic complications | 28 | 5.22E-19 | 2.63E-18 |
| hsa04068 | FoxO signaling pathway | 31 | 1.36E-18 | 6.51E-18 |
| hsa05163 | Human cytomegalovirus infection | 39 | 4.66E-18 | 2.12E-17 |
| hsa05169 | Epstein-Barr virus infection | 36 | 3.92E-17 | 1.71E-16 |

**Table S3.** Top 10 KEGG Pathways for microRNA with differential expression in amnion membrane.

| **ID** | **Description** | **Count** | **P value** | **Q value** |
| --- | --- | --- | --- | --- |
| hsa04151 | PI3K-Akt signaling pathway | 91 | 7.55E-32 | 2.19E-30 |
| hsa04010 | MAPK signaling pathway | 82 | 2.65E-31 | 5.50E-30 |
| hsa04933 | AGE-RAGE signaling pathway in diabetic complications | 48 | 4.11E-31 | 7.47E-30 |
| hsa05161 | Hepatitis B | 60 | 6.18E-31 | 9.98E-30 |
| hsa04068 | FoxO signaling pathway | 52 | 1.28E-28 | 1.55E-27 |
| hsa04668 | TNF signaling pathway | 48 | 7.16E-28 | 7.43E-27 |
| hsa04218 | Cellular senescence | 55 | 3.78E-27 | 3.66E-26 |
| hsa01521 | EGFR tyrosine kinase inhibitor resistance | 39 | 4.85E-26 | 4.15E-25 |
| hsa01522 | Endocrine resistance | 42 | 7.09E-25 | 5.42E-24 |
| hsa05165 | Human papillomavirus infection | 76 | 1.23E-23 | 8.51E-23 |

**Table S4.** Top 10 KEGG Pathways for intersecting microRNAs with differential expression in placenta and amnion.

| **ID** | **Description** | **Count** | **P value** | **Q value** |
| --- | --- | --- | --- | --- |
| hsa04218 | Cellular senescence | 16 | 1.23E-12 | 4.37E-11 |
| hsa05169 | Epstein-Barr virus infection | 16 | 6.57E-11 | 1.49E-09 |
| hsa04151 | PI3K-Akt signaling pathway | 20 | 1.22E-10 | 1.49E-09 |
| hsa01522 | Endocrine resistance | 12 | 1.26E-10 | 1.49E-09 |
| hsa05161 | Hepatitis B | 14 | 3.67E-10 | 3.90E-09 |
| hsa04110 | Cell cycle | 13 | 2.81E-09 | 1.87E-08 |
| hsa05167 | Kaposi sarcoma-associated herpesvirus infection | 13 | 3.65E-08 | 2.04E-07 |
| hsa04068 | FoxO signaling pathway | 11 | 4.46E-08 | 2.37E-07 |
| hsa01524 | Platinum drug resistance | 8 | 4.45E-07 | 1.89E-06 |
| hsa05165 | Human papillomavirus infection | 15 | 5.24E-07 | 2.08E-06 |
