# Supplementary figures and images for: Alterations in MicroRNA and Cytokine Expressions in Placental and Amniotic Tissues of COVID‐19 Affected Pregnant Women
Source: Kaohsiung J Med Sci. 2026 Apr 2:e70207. Online ahead of print. doi: 10.1002/kjm2.70207 (PMC13399688; doi:10.1002/kjm2.70207)

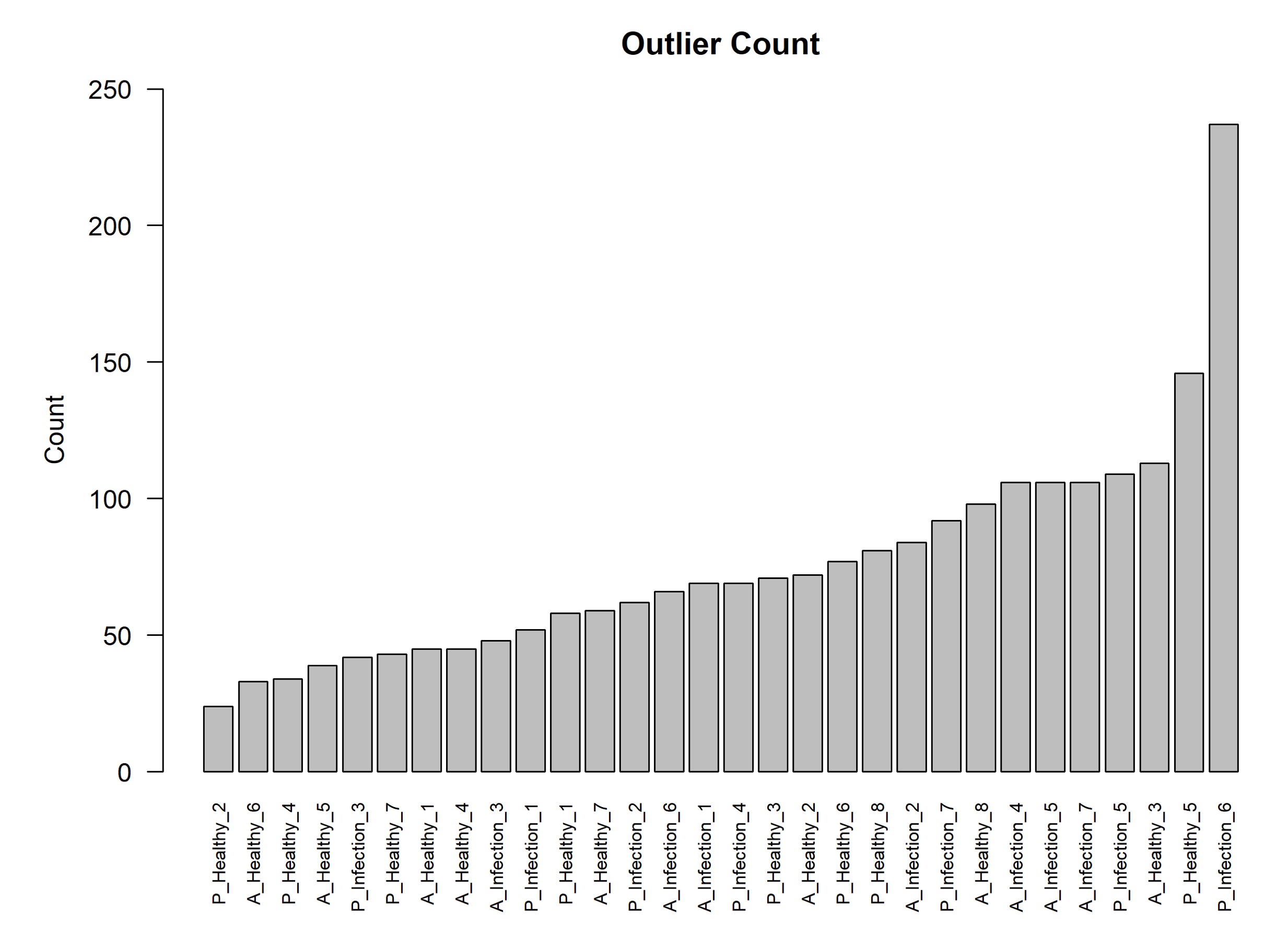

Supplement: Supplementary file 1 — Data S1: Supporting Information. Figure S1: Distribution chart of microRNA outliers. This chart illustrates the number of times each sample is determined to be an outlier for various microRNAs based on the four combinations of placenta, amnion, diagnosed, and healthy. P_infection_6 which is highlighted in red boxes was identified as a significant outlier in both the placental and diagnosed groups. Figure S2: Principal component analysis (PCA) plot of cytokines level from 15 pregnant women. Seven confirmed samples of placenta and amnion tissue and eight healthy samples of placenta and amnion tissue in total. The red boxes in the figure indicate the outlier samples. Among the four outlier samples, two are from amniotic tissue and two from placental tissue. Table S1: Participant demographic and clinical data. Table S2: Top 10 KEGG Pathways for microRNA with differential expression in placental tissue. Table S3: Top 10 KEGG Pathways for microRNA with differential expression in amnion membrane. Table S4: Top 10 KEGG Pathways for intersecting microRNAs with differential expression in placenta and amnion. [file KJM2-9999-e70207-s001.zip › figS1.jpg]

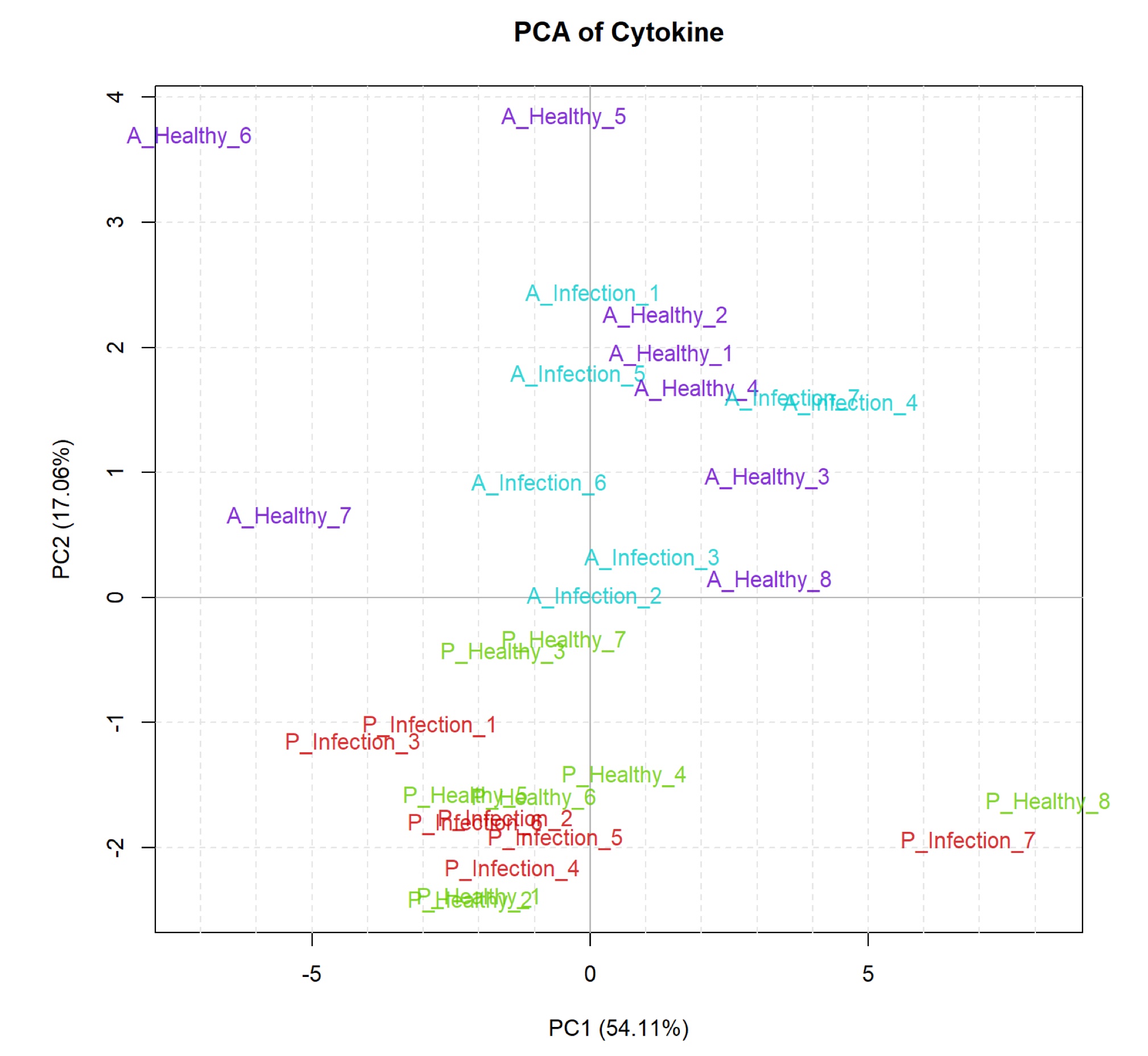

Supplement: Supplementary file 1 — Data S1: Supporting Information. Figure S1: Distribution chart of microRNA outliers. This chart illustrates the number of times each sample is determined to be an outlier for various microRNAs based on the four combinations of placenta, amnion, diagnosed, and healthy. P_infection_6 which is highlighted in red boxes was identified as a significant outlier in both the placental and diagnosed groups. Figure S2: Principal component analysis (PCA) plot of cytokines level from 15 pregnant women. Seven confirmed samples of placenta and amnion tissue and eight healthy samples of placenta and amnion tissue in total. The red boxes in the figure indicate the outlier samples. Among the four outlier samples, two are from amniotic tissue and two from placental tissue. Table S1: Participant demographic and clinical data. Table S2: Top 10 KEGG Pathways for microRNA with differential expression in placental tissue. Table S3: Top 10 KEGG Pathways for microRNA with differential expression in amnion membrane. Table S4: Top 10 KEGG Pathways for intersecting microRNAs with differential expression in placenta and amnion. [file KJM2-9999-e70207-s001.zip › figS2.jpg]
